# Contributions of Intrinsic and Extrinsic Foot Muscles during Functional Standing Postures

**DOI:** 10.1155/2022/7708077

**Published:** 2022-05-05

**Authors:** Sarah T. Ridge, K. Michael Rowley, Toshiyuki Kurihara, Matthew McClung, Jiaxi Tang, Steven Reischl, Kornelia Kulig

**Affiliations:** ^1^Department of Exercise Sciences, Brigham Young University, Provo, UT, USA; ^2^Kinesiology Department, California State University East Bay, Hayward, CA, USA; ^3^Division of Biokinesiology and Physical Therapy, University of Southern California, Los Angeles, CA, USA; ^4^Department of Sport and Health Science, Ritsumeikan University, Kusatsu, Shiga, Japan; ^5^Reischl Physical Therapy, Inc., Signal Hill, CA, USA

## Abstract

**Purpose:**

Maintaining balance during static standing postures requires the coordination of many neuromuscular mechanisms. The role of the intrinsic and extrinsic foot muscles in this paradigm has yet to be clearly defined. The purpose of this study was to explore foot muscle activation during static phases on common weight-bearing tasks of varying loads and balance demands.

**Methods:**

Twenty healthy young adults performed 6 standing postures (single-limb and double-limb stand, squat, and heel raise) with one foot on a force plate. Muscle activity was recorded from the abductor hallucis, flexor hallucis longus and brevis, and tibialis posterior using intramuscular electrodes; surface electrodes were used to record activity from the peroneus longus and tibialis anterior. Two-way repeated measures ANOVA (2 loading conditions × 3 postures) were run to compare muscle activation and center of pressure velocity.

**Results:**

Intrinsic foot muscle activity increased as loading and postural demand increased; however, the specific effects varied for each of the extrinsic foot muscles.

**Conclusions:**

These results suggest that the intrinsic foot muscles play an important role in maintaining static balance. Strengthening intrinsic and extrinsic foot muscles may help increase stability in people who have weak toe flexors or who suffer from a variety of foot pathologies.

## 1. Introduction

Many weight-bearing activities contain static phases where the body experiences posture-dependent loading and balance demands. During these static postures, the foot muscles play a critical role in maintaining foot and ankle positions. However, due to the complex arrangement of the structures within the foot, it is difficult to specifically measure intrinsic foot muscle (IFM) activation. A thorough understanding of how the intrinsic and extrinsic foot muscles (EFM) work together to cope with the load and balance demands during static phases of weight-bearing tasks will allow clinicians, researchers, coaches, and engineers to develop improved injury prevention, treatment, and assistive technologies.

IFM activity increases in response to greater postural demands and in the presence of foot pathologies. Previous research has reported significant activity in the IFMs during single-limb stance (SLS) and bilateral heel raise (BHR), but not during bilateral stance (BLS) in healthy subjects [1, 2]. Kelly et al. [2] suggested that the activation patterns of the quadratus plantae, flexor digitorum brevis, and abductor hallucis (ABDH) muscles during SLS reflect the importance of the IFMs in maintaining balance. Results from a recent study [1] support the idea that the IFMs may stabilize the foot (by stiffening the longitudinal arch and/or the forefoot), while the EFMs are primarily responsible for ankle joint motion and balance control [3]. Although the specific roles of the IFMs during stance have not been determined, these data suggest that altered IFM activation patterns in people with foot and ankle pathologies [4–10] may present stability or balance challenges.

The roles of the EFMs during loading and balance tasks have been documented extensively [3, 11–15]. It is unclear, however, how EFMs and IFMs work together in response to greater postural demands. It may be that IFM activity increases when EFM activity increases. Conversely, the muscle groups may compensate for each other, resulting in decreased activation in one group during increased activity in the other group. One group may respond more to balance demands, while the other responds to changes in magnitude of loading. An IFM and EFM fatiguing exercise protocol resulted in decreased muscle activity and decreased center of pressure (COP) movement during SLS and BLS with eyes closed [16]. Studies that fatigued the EFMs without additional focus on the IFMs reported varied results; some reported increased postural sway during single-limb stance [17–20], though another study reported no change in postural sway [21]. Muscle activity was not reported in most of these studies, and, due to the potentially overlapping functions of the IFMs and EFMs, it is difficult to determine the contribution of individual muscles (or even muscle groups) to these functional outcomes.

Direct measurement during well-controlled tasks is essential to further our understanding of the roles of the IFMs and EFMs in performing daily activities. Since maintaining stability is critical to efficient and safe movement, it is important to document lower leg and foot muscle activity during tasks which challenge stability. Therefore, the purpose of this study was to evaluate IFM and EFM activity during static phases of common functional weight-bearing tasks of varying load and balance demands. We hypothesized that both IFM and EFM activation would be greater during single-limb postures than during the corresponding double-limb postures.

## 2. Methods

### 2.1. Participants

Twenty young adults (7 males, 13 females; age: 27.5 ± 5.6 yrs; height: 170 ± 7.8 cm; weight: 68.3 ± 15 kg) with no history of pain or injury in the lower extremities for the previous six months volunteered for the study and provided informed consent to participate per guidelines by the University of Southern California Health Sciences Campus Institutional Review Board (HS-14-00607). All participants were healthy and recreationally active. The supporting foot was defined as the foot contralateral to the preferred kicking foot.

### 2.2. Instrumentation

Electromyography (EMG) signals were collected from six muscles that are partially responsible for control of the medial longitudinal arch (MLA), toe flexion, ankle plantar flexion, and/or ankle dorsiflexion [22]. Previous research has also suggested their involvement in maintaining balance [2, 23–25]. The muscles included two IFMs (ABDH and flexor hallucis brevis (FHB)) and four EFMs (flexor hallucis longus (FHL), tibialis posterior (TP), peroneus longus (PL), and tibialis anterior (TA)). Four of these muscles (ABDH, FHB, FHL, TP) were instrumented with paired fine-wire intramuscular electrodes (50-*μ*m nickel-chromium alloy wires with nylon insulation with the distal 2 mm stripped of insulation and bent into a hook loaded into 25-gauge 1.5-inch hypodermic needles and sterilized). Insertion techniques were adapted from Perotto [26] and Kelly et al. [27]. In order to ensure the accuracy of electrode placement, ultrasonography (VFX13-5 linear transducer; Sonoline Antares, Siemens Medical Solutions USA, Inc., Malvern, PA) was used to visualize muscle locations. Placement of electrodes was confirmed using mild electrical stimulation and manual muscle testing. The PL and TA were instrumented with surface electrodes (bipolar silver/silver chloride electrodes with an interelectrode distance of 22 mm) placed over the muscles according to guidelines adapted from SENIAM [28]. EMG data were collected using a wireless Noraxon system (Scottsdale, AZ) sampling at 3000 Hz. All of the electrodes were inserted and/or placed only on the supporting foot and lower leg.

Kinematic data were collected at 60 Hz using an 11-camera Qualisys Oqus System (Gothenburg, Sweden). Markers covered in retroreflective tape were placed over the following anatomical landmarks: 1st and 5th metatarsal heads, medial and lateral malleoli, calcaneus, navicular tuberosity, medial and lateral knee joint, and greater trochanter. Ground reaction force and COP data under the instrumented foot were collected with a force plate (Advanced Medical Technology Inc., Watertown, MA) sampling at 1500 Hz. Kinematic, kinetic, and EMG data were synchronized using a common trigger in Qualisys software.

### 2.3. Procedures

While barefoot on the force plate (which provided a stable, firm surface), participants maintained six static standing postures: bilateral stance (BLS), single-limb stance (SLS), bilateral squat (BSQ), single-limb squat (SSQ), bilateral heel raise (BHR), and single-limb heel raise (SHR). During bilateral standing postures, participants were instructed to keep their feet parallel to each other while maintaining a comfortable stance width. Participants were instructed to maintain each position for five seconds with minimal movement. They were offered a light touch using the experimenter's hand if needed; all subjects used a light touch during SHR, 15 out of 20 used a light touch during SSQ, and 10 out of 20 used a light touch during BHR. Each standing posture was performed once in each of three blocks. Within each block, posture order was randomized. The three trials for each standing posture were averaged for analysis. Note that the phase of getting to or leaving the final posture was not included in the analyses; only the static hold in the posture was analyzed. The middle three seconds of the statically held position were analyzed.

### 2.4. Data Processing and Analysis

Surface EMG was bandpass filtered between 30 Hz and 500 Hz and fine-wire EMG between 30 Hz and 1000 Hz. Root mean square (RMS) signal amplitude was calculated over the middle three seconds of the trial and normalized to maximal voluntary isometric contractions (MVIC) recorded during a series of manual muscle tests [29]. Kinematic and kinetic data were low-pass filtered using a fourth-order Butterworth filter with a 6 Hz low-pass cut-off.

COP location was calculated as a percent of foot length from the calcaneus marker to the 1^st^ metatarsal head by dividing the distance between the COP location and the heel by the sum of the distance between the COP location and the heel and the distance between the COP location and the toe. The time series location data were averaged for the duration of the static standing trial. Center of pressure velocity (COPV) was calculated by averaging the frame-by-frame COPV defined as the change in planar COP location divided by the time elapsed between frames.

Two-way repeated measures ANOVA (2 loading conditions × 3 postures) were run to compare muscle activation and COPV during each static posture. Significance was set at *α*  < 0.05. When a significant main effect was found, post hoc comparisons were run with the Holm correction for multiple comparisons. All statistical analyses were run in JASP (JASP [30]).

## 3. Results

**Figure 1:**
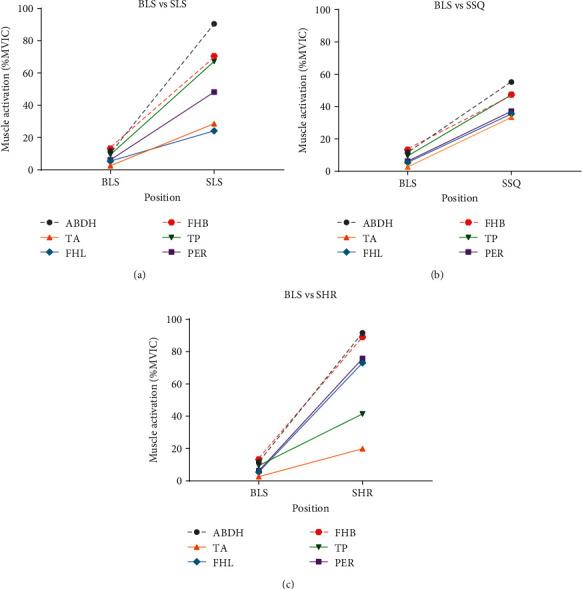
Comparison of average muscle activation during bilateral and single-limb stance positions.

Increased loading (comparing single-limb to double-limb stances) did not result in greater muscle activation in all muscles during all postures (Table 1). While both measured IFMs (ABDH and FHB) and one EFM (PL) were significantly more active during all single-limb positions than during double-limb positions, the responses of the other EFMs varied. For example, the TA was significantly more active during SLS and SHR than during the corresponding double-limb positions but similarly active in SSQ and BSQ (Figures 1(a)–1(c)). The TP was significantly more active during SLS and SSQ than during the corresponding double-limb positions but similarly active in SHR and BHR. Finally, the FHL was only significantly more active during SSQ than BSQ.

**Figure 2:**
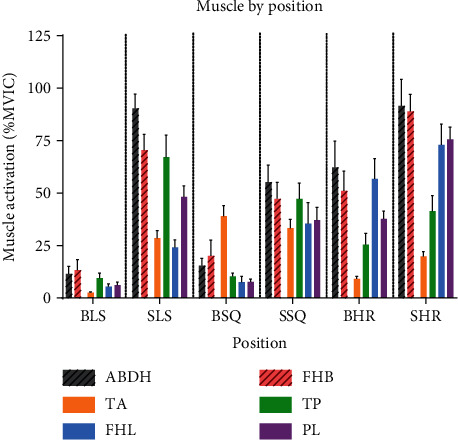
Average muscle activity during each static posture. Error bars represent standard deviation.

The highest levels of muscle activation occurred during the SHR in the ABDH, FHB, FHL, and PL (Figure 2). The TP was most active during the SSQ, while the TA was most active during the BSQ. Interestingly, the BSQ was the only movement in which the TA was significantly more active than any of the other tasks.

COPV was significantly greater during SLS and SSQ than during the corresponding bilateral stance positions. However, COPV was significantly greater during BHR than during SHR.

## 4. Discussion

The purpose of this study was to compare IFM and EFM activity while maintaining three common static standing positions (quiet standing, heel raise, and squat) under two body-weight loading conditions (single-limb and double-limb support). We hypothesized that IFM and EFM activation would be greater during single-limb stances than during the corresponding double-limb stances. We found that generally, muscles were more active during the single-limb positions when compared to the corresponding double-limb positions, although the magnitude of the increases varied between muscles and positions. In addition, our findings suggest that muscles were activated as functional groups, rather than grouped by location (i.e., intrinsic vs. extrinsic). Under the observed conditions, it is apparent that contributions of IFMs and EFMs are important for maintaining a variety of static standing positions.

### 4.1. The Effect of Increased Load between Double-Limb and Single-Limb Static Standing Positions

Research has reported that while IFM show little to no activity during double-limb upright standing [31–33], they are active during single-limb upright standing [2]. EFMs have also shown increased activity during single-limb standing compared to double-limb standing [34]. However, there has been little research done measuring foot muscle activity during variations of upright standing—positions which may be adopted during activities of daily living. These activities, such as climbing stairs, entering/exiting cars, stepping into the shower, using the toilet, or reaching an item on a high shelf, require maintaining periods of static stability while keeping the knee and ankle in concurrently flexed or extended positions.

Our hypothesis that muscle activity would be greater during single-limb positions was met for all muscles except the TA during the squat task (this exception may have been due to the external support participants used during SSQ). Although we could not find comparable reported values (%MVIC) for lower leg muscle activity while maintaining a SSQ position, our data are similar in magnitude during SHR to previous research [35]. The amount of muscle activity change between double-limb and single-limb body-weight load conditions within each task was variable and will be discussed in more detail later in this section.

### 4.2. The Effect of Varied Functional Static Standing Positions

**Figure 3:**
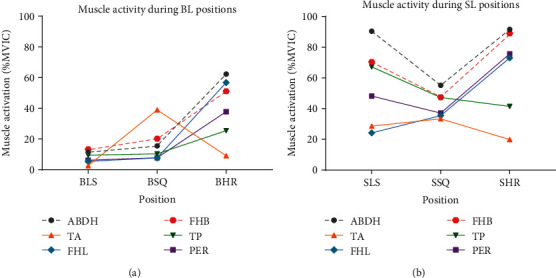
Comparison of average muscle activity among bilateral positions (a) and single-limb positions (b).

**Figure 4:**
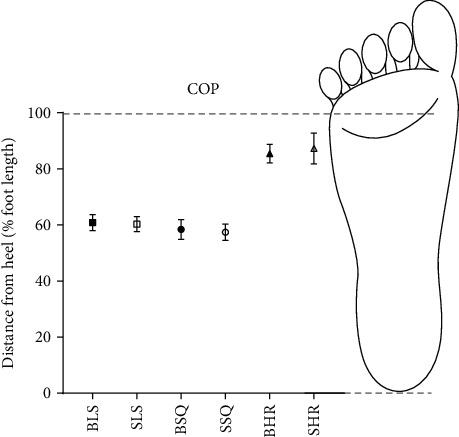
Center of pressure position along the long axis of the foot during each static posture.

In a comparison of double-limb positions, every muscle measured showed greater activation in the squat position than quiet standing (Figure 3(a)). All muscles, except for TA, showed the greatest activation in heel raise position. This increased muscle activation is likely due to increased ankle joint moments compared to the other standing positions. While joint moments were not calculated in this study, the static nature of the positions adopted and the lack of change in loading between the positions allows us to use COP location as a surrogate value. COP location was further anterior in the heel raise condition than in the other two conditions (Figure 4), providing support for the idea that ankle joint moments were higher in the HR positions due to the larger moment arm created by the COP position.

Comparison of muscle activation between single-limb positions is slightly more complicated than for double-limb positions, as muscles were activated differently than in double-limb positions. This is particularly interesting when considering the increased muscle activity during SLS compared to SSQ (Figure 3(b)) as the foot and lower leg are in comparable positions, while knee and hip flexion during SSQ results in a slightly (nonsignificantly) more posterior COP location. While greater muscle activity during SLS than SSQ may seem surprising based on the perceived difficulty of maintaining each position, we believe that this indicates that most of the muscles measured in this study are not those primarily responsible for maintaining stability in a squat position (i.e., the quadriceps, hamstrings, TA, and gastrocnemius) [36–41]. In the current study, TA was the only muscle which showed its highest activation during the SSQ, though the differences between SSQ, SHR, and SLS were not statistically significant.

Both SLS and SHR positions required near maximal activation of some IFMs and EFMs, though the specific muscles which were most activated during each position were different. For example, TP was more active during SLS compared to SHR position. This could be related to TP's role in balance control during single-limb stance and the stabilizing effect it exerts on the foot as it crosses the ankle joint. Research reporting TP muscle activity during walking shows that maximal activity occurs just prior to heel contact and at midstance—both times in which the ankle is in a neutral position, similar to SLS [42]. Conversely, FHL and PL were significantly more active during SHR than SLS. As FHL and PL are both plantar flexors, it is logical that these would be the most active when maintaining the static HR position. TA and TP seem to play a lesser role in maintaining the SHR position as they only reach 20% MVIC and ~40% MVIC, respectively. This is likely because maintaining a heel raise position causes the COP to shift forward which requires activity from other muscles like the gastrocnemius to stabilize that body position [43]. Muscles that support the MLA, PL and ABDH were both very active during SHR. MLA position while the toes are in extension is often attributed to the windlass mechanism. The activity of the PL and ABDH in the current study supports recent research suggesting that foot muscles are likely to be more involved in MLA movement than previously thought [44–46].

### 4.3. The Interaction of Load and Position

#### 4.3.1. Muscle Activation

Using BLS as our baseline/comparison condition, it is clear that maintaining single-limb upright standing, squat, or heel raise positions requires significantly more foot and lower leg muscle activity. For example, muscle activity during SHR and SSQ were significantly greater than during BLS for all measured muscles. As previously mentioned, research has shown that EFMs are active while maintaining a squat position (or during movements involved in ADL such as getting into or out of a car) [38–40]. Though the IFMs were more active during SSQ than during BLS and BSQ, they were less active than during SLS, implying that EFMs and other muscles may be more important for maintaining stability in the squat position, regardless of loading magnitude. However, the fact that most subjects used external support for balance during the SSQ position may also have influenced the amount of IFM activation required to maintain this position.

Finally, increases in muscle activation were seen in all muscles when comparing SLS to BLS. Although FHL showed ~20% more activity during SLS than during BLS, it did not reach statistical significance. In addition to the increase in load from double-limb to single-limb positions, stabilizing on a single leg causes a greater balance challenge which requires more muscle activation [2].

#### 4.3.2. The Relationship between COP and Muscle Activation during Standing Postures

One way of quantifying balance is by measuring magnitude of COP movement or postural sway. It has been suggested that the IFMs stabilize the foot and control postural sway [3]. However, if the IFMs are not strong enough to maintain balance, the EFMs are used to create larger forces to stabilize the foot [25]. Furthermore, Ferrari et al. [1] concluded that the increased movement of the COP during balance must be predominantly driven by excitation patterns of muscles extrinsic to the foot. The increase in muscle activity in single-limb positions compared to BLS for almost all muscles (both IFMs and EFMs) in the current study support these findings from previous research.

COPV is another metric often used to quantify balance. COPV was significantly greater in SLS than during the baseline BLS position (which showed the lowest COPV value; Table 1). COPV data, while useful, may be limited for interpretation of stability. Higher COPV values in healthy subjects, rather than indicating instability, may actually represent a response for maintaining postural control, as subjects explore the location of their body in space in an attempt to find the most stable position [47]. Given our analysis of intrasubject COPV data, we believe our comparisons between SLS and BLS are still useful. Comparisons between COPV during other double-limb and single-limb positions in this study are complicated by external support used by a number of participants during SHR, SSQ, and BHR positions. It should be noted, however, that although we observed lower COPV during SHR than SLS (probably due to external support), increased muscle activation of the FHL, PL, ABDH, and FHB in the SHR position implies the importance of these muscles for maintaining balance.

### 4.4. Functional Groups

As we simultaneously compare the effects of both increasing loading and maintaining different positions, we see that muscles work together in functional groups rather than groups based strictly on anatomical location (i.e., IFMs and EFMs). These functional groups show activation during different static positions regardless of whether the support was double-limb or single-limb. Hunt et al. [18] made similar conclusions regarding the role of the EFMs during walking. In the current study, every position showed increased activation of the two IFMs measured and at least one EFM. These results support the idea that IFM and EFM groups coordinate activity when shifting from double-limb to single-limb tasks.

### 4.5. Clinical Applications

Our data supports the practice of designing prevention and rehabilitation interventions that focus on the patients' ability to perform specific functions rather than activating specific muscles. Our results show that the activation of both IFMs and a variety of EFMs occurred in some double-limb and single-limb static postures.

Weaker toe flexors [48] have been associated with increased fall risk in older adults [49, 50] and several pathological conditions such as plantar fasciopathy, hallux valgus, pes planus, and claw toe deformity [51–53]. Among other stabilizing functions, fatigue of the ankle plantar flexor muscles has also been associated with impairment of postural control [54]. Magnitude of loading should be considered in addition to the type of movement. For example, when determining which activities should be used for strengthening, clinicians may start with practicing double-limb postures, which will generally require less strength. Our results showed increased muscle activation during BSQ and BHR, which indicates their ability to increase demand on the muscles without presenting as much of a balance challenge. Stability during a variety of single-limb postures should be a subsequent goal, with the ultimate goal of stability during single-limb dynamic movements. Progressing through this series of postures may allow patients' to safely perform a variety of functional movements such as ascending and descending stairs, stepping into and out of the shower, reaching for an overhead object, and picking up an object on the floor.

### 4.6. Limitations

Some limitations of this research should be noted. First, the participants in this study were healthy young adults. While this is a relatively typical population to use in research, particularly when intramuscular EMG is used, there may be limits to the applicability of our findings to older adults or people with foot pathologies. Using this study for comparison, further research should be conducted on other populations. Second, the use of MVICs for the normalization of EMG data may present a limitation. It is difficult to obtain a true MVIC of some of the muscles included in this study. If this was the case, our muscle activation values during the postures of interest may be exaggerated. However, our values are comparable to the few reported in the literature which were also normalized to participants' MVICs.

Finally, as previously mentioned, the fact that subjects used external support during the more difficult stance tasks (BHR, SSQ, and SHR) may present a limitation. It is difficult to assess how much this procedure may have confounded our results. In future studies of this nature, an instrumented device should be used to capture and quantify the magnitude of support used during challenging stance tasks. Keeping this consistent between standing conditions, even in conditions where participants may not need the support, would allow for more accurate comparisons. This is important due to the large impact a low-magnitude light-touch has on balance dynamics [55].

## 5. Conclusion

The current study is one of only a few profiling studies that have reported intrinsic and extrinsic foot muscle activity during static postures other than upright standing. As expected, our data show more muscle activity during single-limb support positions than during double-limb support positions. The magnitude of the increase in muscle activity varied by muscle and was not necessarily associated with whether the muscle was an intrinsic or extrinsic foot muscle. These findings help further our understanding of the function of intrinsic foot muscles and may influence foot and ankle injury prevention and rehabilitation practices.

## Figures and Tables

**Table 1:** Muscle activation (%MVIC) and COPV (cm/s) data for each posture and loading condition. All data are represented as average ± standard deviation.

Muscle	Load	Posture			Main effects		
		Stand	Squat	Heel raise	Load	Posture	Interaction
ABDH	BL	11.4 ± 15.8	15.5 ± 15.2^a^	62.3 ± 56.2^a,b^	<0.001∗	<0.001∗	0.002∗
		•	•	•			
	SL	90.5 ± 77.9^#^	55.2 ± 36.5^a,#^	91.5 ± 56.2^b,#^			

FHB	BL	13.2 ± 22.9	20.3 ± 33.5^a^	51.1 ± 42.2^a,b^	<0.001∗	<0.001∗	0.035∗
		•	•	•			
	SL	70.6 ± 57.5^#^	47.3 ± 34.6^#^	89.0 ± 75.1^b,#^			

TA	BL	2.6 ± 1.9^b^	39.0 ± 23.0^a^	9.1 ± 5.5^b^	<0.001∗	<0.001∗	<0.001∗
		•		•			
	SL	28.7 ± 15.7^#^	33.5 ± 18.5^#^	19.9 ± 10.4^b,#^			

TP	BL	9.5 ± 10.8	10.3 ± 6.8	25.5 ± 24.3	<0.001∗	0.236	0.003∗
		•	•				
	SL	67.2 ± 61.5^#^	47.4 ± 33.6^#^	41.3 ± 33.0^a,#^			

FHL	BL	5.6 ± 5.6	7.6 ± 12.0	56.8 ± 43.7^a,b^	0.001∗	<0.001∗	0.463
			•				
	SL	24.2 ± 16.2	35.4 ± 44.8^#^	73.0 ± 44.5^a,b,#^			

PER	BL	6.2 ± 6.3	7.7 ± 5.4	37.9 ± 16.0^a,b^	<0.001∗	<0.001∗	0.085
		•	•	•			
	SL	48.1 ± 23.8^b,#^	37.1 ± 27.8^a,#^	75.7 ± 26.3^a,b,#^			

COPV	BL	1.31 ± 0.48	1.66 ± 0.65	2.56 ± 1.37^a,b^	<0.001∗	0.150	<0.001∗
		•	•				
	SL	4.23 ± 1.09^b,#^	3.35 ± 1.59^a,#^	2.13 ± 0.95^a,b,#^			

∗ = significant *p*-value. • = significant difference between load conditions. *a* = significant difference from stand (same amount of load). *b* = significant difference from squat (same amount of load). # = significant difference from BLS.

## Data Availability

The data used to support the findings of this study are available from the corresponding author upon request.

## Abbreviations

ABDH: Abductor hallucis
ANOVA: Analysis of variance
BHR: Bilateral heel raise
BLS: Bilateral stance
BSQ: Bilateral squat
COP: Center of pressure
COPV: Center of pressure velocity
EFM: Extrinsic foot muscle
EMG: Electromyography
FHB: Flexor hallucis brevis
FHL: Flexor hallucis longus
IFM: Intrinsic foot muscle
MVIC: Maximal voluntary isometric contractions
PL: Peroneus longus
RMS: Root mean square
SLS: Single-limb stance
SSQ: Single-limb squat
SHR: Single-limb heel raise
TA: Tibialis anterior
TP: Tibialis posterior.
